# The job demands-resource model and performance: the mediating role of employee engagement

**DOI:** 10.3389/fpsyg.2023.1194018

**Published:** 2023-06-22

**Authors:** Da Ye Lee, Yunseong Jo

**Affiliations:** ^1^College of Liberal Arts, The University of Suwon, Hwaseong, Republic of Korea; ^2^Social Science Korea Research Team, Chung-Ang University, Seoul, Republic of Korea

**Keywords:** job autonomy, psychological well-being, personal initiative, employee engagement, prosocial behavior, fully mediated model

## Abstract

In case of a major social crisis, such as the coronavirus pandemic, the most important measure is to identify the determinants influencing employee health and well-being, which are directly linked to workplace job performance. Many studies have explored the role of employee engagement in the relationship between job resources, psychological capital, and job performance; however, only a few have investigated the relationships reflecting rapid changes in the work environment represented by digital transformation and a major social crisis. Considering this, this study examines how job autonomy and psychological well-being, which lower employee anxiety about health and welfare, influence in-role performance in the form of proactive employee characteristics as well as extra-role performance in the form of prosocial behavior, as mediated by employee engagement. The results of the data analysis of 1,092 corporate employees in Korea supported this model. Specifically, job autonomy and psychological well-being influence job performance (i.e., personal initiative and prosocial behavior) through improvements in employee engagement. Based on these findings, the study also discusses the implications of the results, future directions, and limitations.

## 1. Introduction

Owing to the Fourth Industrial Revolution, business environment has been undergoing rapid economic and technological changes. Furthermore, future development and growth can no longer be guaranteed, given the use of strategies that focus only on short-term profit creation (Schwab, 2016). The recent outbreak of the coronavirus disease (COVID-19) has accelerated these changes. Organizations face increasingly fierce competition, even as the pandemic (similar to previous crises) has increased ambiguity and uncertainty in the business environment, compelling organizations to undertake measures to protect the health of employees and improve the chances of organizational survival (Combe and Carrington, 2015). Just as the pandemic forced political leaders to take measures at the national level, it also pushed families, individuals, and leaders of organizations to take measures at their respective levels (Kniffin et al., 2021). In a major social crisis such as COVID-19, the most important measure is to identify the determinants influencing employee health, well-being, and organizational survival, which are directly linked to performance in the workplace.

Under these circumstances, organizations must cope with unpredictable changes in the management environment and establish specific strategies to survive and thrive (Haas and Yorio, 2018; Sherman and Roberto, 2020). Similarly, effective management of human resources is emphasized (Kim, 2016) as a core element in maintaining competitiveness and maximizing organizational performance. As human resources are regarded as the main driver of performance, organizations are investing in them and making strong efforts to manage them more effectively and develop employee competence (Haas and Yorio, 2018; Sherman and Roberto, 2020). Organizations are trying to develop the resources required to change the behaviors of their members and improve job performance (Salanova and Schaufeli, 2008), as well as provide support and management for employees to perform their job effectively and improve performance.

During a crisis, it is important for organizations to facilitate rapid responses and self-initiated changes among employees through support and management (Demerouti and Bakker, 2023). Accordingly, it is necessary to consider employee-related resources to investigate measures to improve organizational performance (Kwon and Kim, 2020). There are two main resources to consider: job resources, which play a motivational role and provide support for employees to achieve work goals; and personal resources, which refer to an individual’s sense of their ability to control and influence their environment successfully (Hobfoll et al., 2003; Bakker and Demerouti, 2008).

First, job resources must be considered as factors that influence job performance. Job performance is determined not only by personal factors but also by employees’ work environment, authority, autonomy, and support from leaders (Hackman and Oldham, 1975). A high level of job resources offsets the negative effects of job environment and is instrumental in achieving job performance. More importantly, job resources improve employees’ job engagement, which leads to high job performance (Macey and Schneider, 2008; Demerouti and Cropanzano, 2010).

A substantial body of research exists on psychological capital, a variable of employees’ personal resources that is closely related to job performance (Larrabee et al., 2010; Seo et al., 2014). In the field of human resources, psychological capital is critical for promoting engagement and improving job performance (Demerouti and Bakker, 2023). Employees with high levels of positive psychological capital tend to show fewer negative factors, such as burnout and job turnover, while showing a greater number of positive factors, such as job satisfaction, engagement, and job performance (Seo et al., 2014; Laschinger et al., 2015; Zhao et al., 2015). Thus, positive psychological capital facilitates job engagement, motivating employees to achieve job goals and positively influencing job performance (Luthans, 2002; Sweetman and Luthans, 2010).

Engagement has been studied as a core variable mediating the relationship between job resources, psychological capital, and job performance (Bakker, 2009; Kim, 2017; Kim et al., 2018). It is perceived as an important research subject by practitioners and researchers because it is related to performance variables, such as innovative behavior, productivity, and stability (Bakker and Leiter, 2010; Zhong et al., 2016). It has been found to be highly correlated with outcomes such as performance, creativity, and health while increasing job productivity and maintaining employee well-being (Bakker and Demerouti, 2008).

Despite a large number of studies exploring the role of employee engagement in the relationship between job resources, psychological capital, and job performance, few studies have investigated the relationships between job resources, psychological capital, employee engagement, and job performance all together. Previous studies have investigated the effects of job resources on employee engagement or job performance (Schaufeli and Bakker, 2004; Rothmann and Joubert, 2007), the effect of psychological capital on employee engagement or job performance (Luthans, 2002; Bandura and Locke, 2003; Kim, 2016), and the effect of employee engagement on job performance (Shuck and Wollard, 2010). However, most of these explored partial relationships between variables, rather than examine the structural relationship between major variables, as we attempt in this study. Additionally, the majority of research on employee engagement has focused on job or work engagement. Most prior studies have used the Utrecht Work Engagement Scale (UWES) to measure work engagement (Bailey et al., 2017; Saks et al., 2022). As a result, results were narrowly related to job and work engagement. However, recently, employee engagement is recognized as a multidimensional concept. For example, employee has the various roles such as “a work role and a role as a member of their organization” (Song et al., 2021; Saks et al., 2022). Thus, we aim to investigate the relationship between the variables influenced by rapid changes in the work environment represented by digital transformation, while also considering the effects of the COVID-19 pandemic as a major social crisis. We examine how job autonomy (Demerouti and Bakker, 2023) and psychological well-being (Rudolph et al., 2021; Demerouti and Bakker, 2023), which lower employee anxiety about health and welfare, influence in-role performance in the form of proactive employee characteristics, as well as extra-role performance in the form of prosocial behavior, as mediated by employee engagement.

This study contributes to a better understanding of the importance of employees’ positive emotions and proactive behaviors in times of crisis characterized by fast-paced changes to the business environment; it makes theoretical and practical contributions toward guiding the management and developing human resources to improve work efficiency in organizations.

## 2. Relationship between job autonomy and employee engagement

Autonomy is the degree to which a job gives employees discretion while working to control the work process on their own terms (Morris and Feldman, 1996). Furthermore, it allows employees to determine how to do their job (including the selection of tools and instruments for planning and establishing the work process), and to take responsibility for the results (Amabile et al., 1996).

Job autonomy is also defined as the degree of freedom, independence, and discretion in decision making and skill application in one’s work, including discretion in determining the time schedule for work and work procedures (Kim et al., 2018). As autonomy gives employees a sense of control over their work and work responsibilities, it positively influences their job satisfaction and sense of achievement (Rhee et al., 2017). The degree of autonomy may differ according to the organization. Autonomy can be linked to motivation and passion for work, and a lack of autonomy may lead to lower job performance in specific areas. The range of autonomy includes task methods, composition of the task team, scope of the task, and goal setting (Wall et al., 1995). Job autonomy also refers to the degree of control that employees have to select a job for themselves and adjust the speed of work while performing the job. This can increase overall job satisfaction and improve and develop employee attitudes toward job performance (Dodd and Ganster, 1996). As an important motivational factor, job autonomy influences employees’ attitudes (Deci and Ryan, 2000). Job autonomy is also a major predictor of organizational and individual job performance (Hackman and Oldham, 1976; Campion, 1988) and has been used as an independent variable that influences parameters and dependent variables in many previous studies. It is also known to affect the psychological status of an individual, eliciting positive work-related outcomes (Hackman and Oldham, 1975, 1976).

Kahn (1990), who was widely credited with the first application and use of engagement theory to the workplace, defined engagement as ‘the harnessing of organization members’ selves to their work role; in engagement, people employ and express themselves physically, cognitively, and emotionally during role performances’ (p. 694). Based on the concept of personal engagement, as defined by Kahn (1990), follow-up studies have taken various approaches and used a range of terms, including personal engagement, job engagement, work engagement and employee engagement, and the concept of engagement has been presented from different perspectives (Leiter and Bakker, 2010; Shuck, 2011). In the early 2000s, the rise of positive psychology sparked discussions related to happiness and mental well-being experienced at work and the concept of work engagement was studied in relation to employee burnout and well-being (Leiter and Maslach, 2010). For example, work engagement refers to “a positive, fulfilling, work-related state of mind that is characterized by vigor, dedication, and absorption” (Schaufeli et al., 2002, p. 74). Recently, the concept of engagement has been examined engagement from both the job and organizational perspectives. Hence, to emphasize that the individual belongs to an organization, the word ‘employee’ was added to the term ‘engagement’ (i.e., employee engagement). Actually, Shuck et al. (2017) defined employee engagement as “an active, work-related positive psychological state operationalized by the intensity and direction of cognitive, emotional, and behavioral energy” (p. 959).

Breaugh and Becker (1987) identified job performance, commitment, employee engagement, and autonomy as elements of job attitude that elicit organizational engagement. Beer et al. (1985) argued that perceived control reduces employees’ interest in their job and increases turnover, while job autonomy reduces turnover and improves motivation and job performance. Miller (1990) found that job autonomy has a positive effect on employees’ job satisfaction, stress reduction, and job performance. Schaufeli and Bakker (2004) confirmed the positive effects of job resources such as feedback, autonomy, and task significance on job engagement. Job resources such as skill variety, task significance, and autonomy are crucial factors in employee job performance. According to a study by the Ministry of Education in Helsinki, teachers who lacked job resources showed lower job performance and higher burnout and dropout rates (Hakanen et al., 2006). One study that examined job resources classified into job control and support from leaders and colleagues, found that when more control over their job is given to employees, they are likely to feel a greater sense of responsibility in performing their job leading to higher employee engagement (Binnewies et al., 2008; Karatepe et al., 2013). Job resource factors influencing employee engagement, job autonomy, task diversity, task significance, performance feedback, social support, and the work environment have been identified (Macey and Schneider, 2008). Job resources, such as feedback, autonomy, and learning opportunities, are not only necessary to meet job demands, but also to determine employee engagement (Broeck et al., 2010; Halbesleben, 2010).

*H1*: Perceived autonomy support has a positive effect on employee engagement.

## 3. Relationship between psychological well-being and employee engagement

Psychological well-being is defined using many terms, including happiness, quality of life, life satisfaction, subjective well-being, psychological health, sense of well-being, and well-being. This term first appeared in academic literature as “positive mental health” (Jahoda, 1959). Jahoda identified the following six sub-components of positive mental health: (1) self-acceptance, (2) effort of growth and self-actualization, (3) integration of personality, (4) autonomy from social pressure, (5) perception of reality, and (6) environmental mastery. It is a closely related concept with psychological capital (Manzano-García and Ayala, 2017), so two concepts have been compared with each other. The four characteristics of psychological capital, such as the employee’s willingness and methods to achieve their goals, the optimism about reaching realistically positive outcomes, the confidence to make a positive difference in their work environment, and the resilience to quickly recover from setbacks, have a very close relationship with the theoretical basis. In other words, the integration of hope, efficacy, resilience, and optimism, which are the core of psychological capital, is an important antecedent as available resources and mechanisms to promote well-being (Youssef-Morgan and Luthans, 2015). Youssef-Morgan and Luthans (2015) found that domain-specific satisfaction led to higher overall psychological capital, which led to higher overall well-being. Therefore, positive assessments and comprehensions of circumstances can be influenced by psychological capital, which in turn can affect well-being (Chawla and Sharma, 2019).

Empirical studies on psychological well-being, job satisfaction, organizational commitment, and psychological capital have found a strong positive relationship between self-efficacy and many job-related outcomes and perceived as an important factor in psychological capital (Schaufeli and Bakker, 2004). These personal resources are positive self-reflections related to resilience and other factors, and they are highly related to individual abilities and senses (Hobfoll et al., 2003). Employees with high self-efficacy are more interested in and passionate about their job, which improves job performance (Bandura and Locke, 2003). Previous studies on psychological capital among nurses have confirmed that those with high self-efficacy are likely to have lower job stress, burnout, and turnover intentions and higher job satisfaction, employee engagement, and job performance (Duggleby et al., 2009; Salanova et al., 2011; Federici and Skaalvik, 2012; Laschinger et al., 2015; Zhao et al., 2015). Also, Sharma and Sharma (2015) contend that employees with psychological well-being felt positive self-efficacy and engaged with their work. On the contrary, when employees get burn out, they manifest signs of exhaustion and become less engaged at work.

In a study on psychological capital, employee attitudes, and behavioral outcomes, Youssef-Morgan and Luthans (2007) reported that psychological capital has a positive effect on job happiness, job satisfaction, and engagement among employees. Gong et al. (2019), through the study of enterprises, found that psychological capital can have a positive impact on job performance and burn out. Salanova et al. (2011) demonstrated that employee self-efficacy improves positive emotions, which influences employee engagement. It has also been found that employees’ perceived psychological atmosphere influences employee engagement, which in turn influences performance, mediated by effort (Bakker and Oerlemans, 2016).

*H2*: Psychological well-being has a positive effect on employee engagement.

## 4. Relationship between employee engagement and performance

One problem with the literature on the effects of engagement on performance is that the traditional definition of performance is broad and can be interpreted in various ways (Demerouti and Cropanzano, 2010). Job performance is the result of employees playing a role in achieving organizational goals and tasks. Job performance is a matter of widespread concern as a factor required for the operation of efficient organizations (Miller, 1990). In general, job performance refers to the degree to which an employee’s job is performed successfully, akin to the definition of productivity used by industrial psychologists (Pinous, 1986).

Demerouti and Cropanzano (2010) classified job performance into in- and extra-role performance. In-role performance is defined as behavior that directly supports the formally requested outcomes and organizational goals while performing a job. In-role performance, however, emphasizes the means of personal performance toward an organizational goal. A key factor is personal initiative. Extra-role performance refers to an employee’s discretionary behavior as a member of an organization, including prosocial behavior. It is believed that discretionary behavior does not necessarily influence the productivity of employees but facilitates the effective functioning of the organization (Demerouti and Cropanzano, 2010). Studies on the consequences of employee engagement on performance suggest that employees with high levels of employee engagement belong to high-performing groups (Harter, 2001; Kwon and Kim, 2020). In contrast, employees with high levels of engagement tend to proactively increase their job resources and are involved in innovative and proactive behaviors for better performance (Wrzesniewski et al., 1997; Saks, 2006). In a study conducted among nurses, Nasurdin et al. (2018) found that employee engagement positively influences job performance.

*H3*: Employee engagement has a positive effect on personal initiative.

*H4*: Employee engagement has a positive effect on prosocial behavior.

## 5. The mediating role of employee engagement

**Figure 1:**
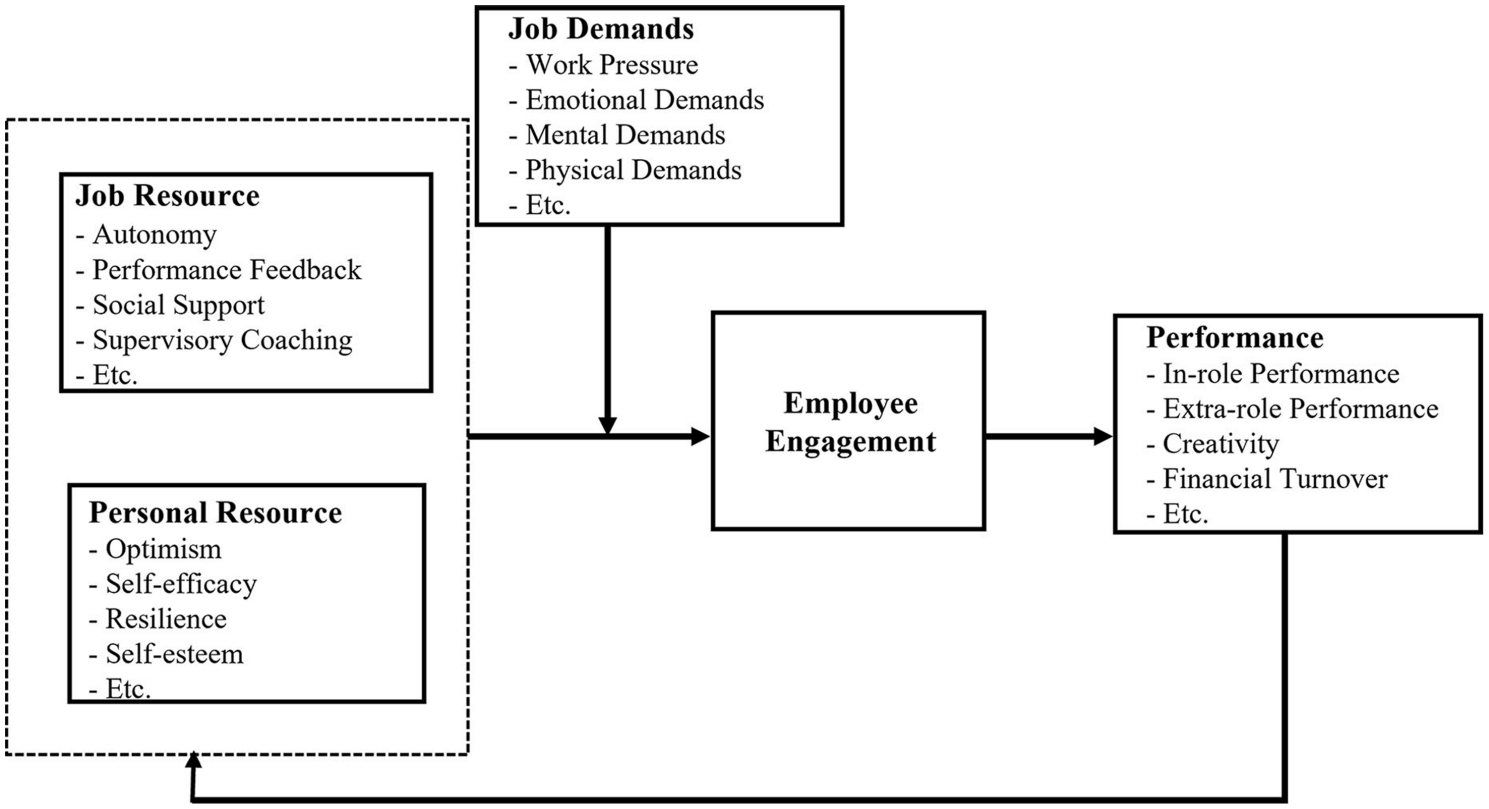
The original JD-R model of engagement (Bakker et al., 2007).

The job demands-resources (JD-R) model–the theoretical foundation of this study, is an integrated model presented to explain job burnout and employee engagement among organizational employees, using the theory of positive and negative outcomes at the personal level (Demerouti et al., 2001). Several studies, which developed the concept of the JD-R model, found that there are personal resources based on personal characteristics, in addition to job resources, which influence employee engagement (Bakker and Demerouti, 2008; Bakker and Leiter, 2010). Also, job demand, as the factor that reduces engagement, is defined as aspects of the job that require sustained physical or mental effort, such as emotional demands and unfavorable work conditions (Schaufeli and Tarris, 2014; Figure 1).

**Figure 2:**
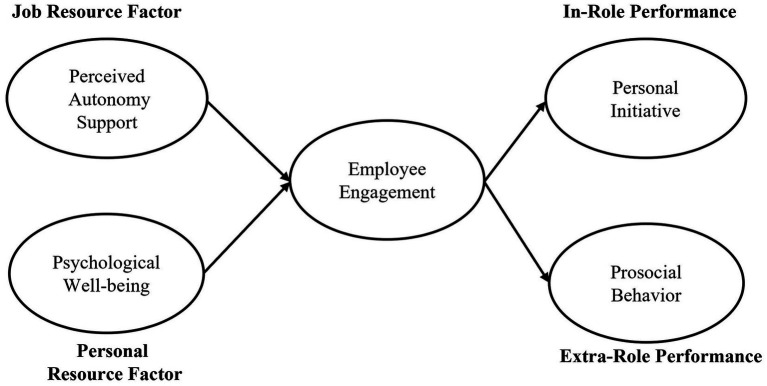
Research framework.

According to the JD-R model, employee engagement is facilitated by job resources and personal resources (Schaufeli and Bakker, 2004) and produces positive outcomes such as innovative behavior (Kwon and Kim, 2020). Schaufeli and Tarris (2014) described employee engagement as characterized by immense spirit and psychological flexibility while working (vigor), strong feeling of importance, keen interest, devotion, and challenging working tasks (dedication), and highly concentrated and cheerful preoccupation with one’s work (absorption). Previous research on employee engagement showed that engaged members tend to use the resources they have to be more productive and possess the skills and energy for the job (Bakker and Demerouti, 2008; Demerouti and Cropanzano, 2010; Bakker and Oerlemans, 2016; Kwon and Kim, 2020). According to broaden-and-build theory (Fredrickson, 1998), positive emotions by engagement experience serve to broaden an individual’s thought–action repertoire, which in turn has the effect of building that individual’s physical, intellectual, and social resources to facilitate in-role performance and extra-role performance. The results of these studies confirm that employees who are energetic, devoted, and focused on their jobs of their own volition are likely to show high performance. Based on this discussion, the following hypotheses were formulated (Figure 2).

*H5*: Employee engagement mediates the effect of perceived autonomy support on personal initiative.

*H6*: Employee engagement mediates the effect of perceived autonomy support on prosocial behavior.

*H7*: Employee engagement mediates the effect of psychological well-being on personal initiative.

*H8*: Employee engagement mediates the effect of psychological well-being on prosocial behavior.

## 6. Materials and methods

### 6.1. Participants/ethical consideration

We collected data through an online survey of private enterprises in South Korea, using a convenience sampling method. All respondents received information about the research goal, assurances about the privacy of their answers, and information about their right to withdraw their consent at any time. Participants received an online gift card. Regarding ethical matters, the survey was conducted in accordance with the APA’s principles on research ethics. Twenty-three careless responses were excluded to ensure the validity of the survey results. Subsequently, the analyzes were conducted on a final pool of 1,092 valid responses. Table 1 showed that the result of frequency analysis of the participants.

**Table 1:** Participants characteristics (*N* = 1,092).

Category		Frequency	Percentage
Gender	Male	544	49.8
	Female	548	50.2
Age	20s	272	24.9
	30s	270	24.7
	40s	275	25.2
	50s	275	25.2
Industry type	Manufacturing	232	21.2
	Human health and social work activities	142	13.0
	Personal services	125	11.4
	Wholesale and retail trade	97	8.9
	Professional, scientific, and technical activities	87	8.0
	Information and communications	86	7.9
	Construction	83	7.6
	Business facilities management and business support services	49	4.5
	Financial and insurance activities	37	3.4
	Transportation	22	2.0
	Others	132	12.1
Job type	Financial accounting	177	16.2
	Customer service	161	14.7
	Human resource management and development	134	12.3
	Management planning	123	11.3
	Production	118	10.8
	Sales and marketing	93	8.5
	General affairs	76	7.0
	Trade	34	3.2
	Others	176	16.1
Position	Staff members	378	34.6
	Assistant managers	256	23.4
	Senior managers	214	19.6
	Deputy general managers	88	8.1
	General managers	111	10.2
	Executives	44	4.0
Total		1,092	100.0

### 6.2. Measures

Because the measurement tools used in this study were developed in English, they were translated into Korean. The translation was validated by two bilingual professors. Then, the Korean version was translated back into English for comparison with the original scale. Furthermore, all items were measured using a 5-point Likert scale ranging from 1 (strongly disagree) to 5 (strongly agree).

#### 6.2.1. Perceived autonomy support

Perceived autonomy support was assessed using the Work Climate Questionnaire (Baard et al., 2000). The items measured employees’ perception of the degree of autonomy supportiveness of their managers using a 6-item scale (see selfdeterminationtheory.org). An example of the item is as follows: “I feel that my manager provides me choices and options.” Cronbach’s alpha coefficient was 0.93. The overall fit of perceived autonomy support met the cut-off criteria (*x*^2^ = 203.737, df = 9, TLI = 0.94, CFI = 0.96, RMSEA = 0.14, SRMR = 0.03).

#### 6.2.2. Psychological well-being

Psychological well-being was assessed using a scale developed by Ryff and Keyes (1995). Cronbach’s alpha for the scale coefficient was 0.79. The scale included six dimensions: autonomy (i.e., “I tend to be influenced by people with strong opinions”), environmental mastery (i.e., “I am good at managing the responsibilities of daily life”), personal growth (i.e., “For me, life has been a continuous process of learning, changing, and growth”), positive relations with others (i.e., “Maintaining close relationships has been difficult and frustrating for me”), purpose in life (i.e., “Some people wander aimlessly through life, but I am not one of them”), and self-acceptance (i.e., “I like most parts of my personality”). A total of 18 items were grouped into three dimensions. The analysis of the measurement model showed good fit indices (*x*^2^ = 22.767, df = 6, TLI = 0.97, CFI = 0.99, RMSEA = 0.05, SRMR = 0.02).

#### 6.2.3. Employee engagement

The employee engagement scale to assess employee engagement was developed by Shuck et al. (2017). The scale’s psychometric properties have been satisfactorily evaluated in past studies using samples of Korean employees (Park et al., 2021). The scale includes three sub-dimensions (i.e., “emotional engagement,” “behavioral engagement,” and “cognitive engagement”). Emotional engagement includes four items, such as “I care about the future of my company.” Behavioral engagement includes four items, such as “I am willing to put in extra effort without being asked.” Cognitive engagement includes four items, such as “I am really focused when I am working.” Cronbach’s alpha coefficient was 0.92. The measurement model analysis revealed good fit indices (*x*^2^ = 291.655, df = 51, TLI = 0.96, CFI = 0.97, RMSEA = 0.07, SRMR = 0.03).

#### 6.2.4. Personal initiative

Personal initiative refers to a behavioral syndrome that develops long-term goals and implements one’s ideas. The Personal Initiative Scale was derived from Frese et al. (1997), which has also been validated among Korean employees (Cho et al., 2007). The scale contains seven items, such as “I am particularly good at realizing ideas.” Cronbach’s alpha coefficient was 0.86. Good fit indices were found in the measurement model analysis (*x*^2^ = 33.658, df = 10 TLI = 0.99, CFI = 0.99, RMSEA = 0.05, SRMR = 0.02).

#### 6.2.5. Prosocial behavior

Prosocial behavior assesses employees’ behaviors intended to benefit the organization using seven items developed by McNeely and Meglino (1994). The scale includes items such as “I take action to protect the organization from potential problems.” Cronbach’s alpha coefficient was 0.83. The measurement model analysis revealed that the fit indices were good (*x*^2^ = 31.277, df = 10, TLI = 0.98, CFI = 0.99, RMSEA = 0.04, SRMR = 0.02).

#### 6.2.6. Control variables

Respondents’ demographics, age (Warr and Fay, 2001; Rosi et al., 2019), job type (De Dreu and Nauta, 2009), and industry type (Hahn et al., 2012) were used as control variables in this research, as these play an important role in varying both personal initiative and prosocial behavior.

### 6.3. Data analysis

To test the hypotheses, data analyzes were conducted using SPSS Statistics v.25 and SPSS AMOS v.25. First, SPSS Statistics was used to analyze the descriptive statistics and Pearson’s correlation matrix of all study variables. Second, we conducted a Confirmatory Factor Analysis (*CFA*) using IBM SPSS AMOS to examine the construct validity and reliability. Hu and Bentler’s (1999) index guidelines were used to evaluate the goodness-of-fit indices: TLI and CFI ≥ 0.90, and RMSEA and SRMR ≤ 0.08 were considered indicative of good model fit (Byrne, 2013). Finally, the direct and indirect effects of the variables were analyzed. Regression coefficients and bias-corrected 95% confidence intervals (CIs) were calculated using bootstrapping (5,000 re-samplings). Statistical significance was set at *p* < 0.05. Furthermore, *CFA* for the single common factor model was used to assess common method bias (Podsakoff et al., 2003). The *CFA* result indicated that it fit poorly with the collected data (*x*^2^ = 4367.332, df = 360, TLI = 0.72, CFI = 0.72, RMSEA = 0.10, SRMR = 0.10). As there was no single common factor explaining the major variance, common method bias was not considered a major problem in this study.

## 7. Results

### 7.1. Descriptive statistical analysis

Table 2 presents the basic statistical information and correlation coefficients of the control and research variables. Specifically, the correlations between perceived autonomy support, psychological well-being, employee engagement, personal initiative, and prosocial behavior reached a significant level (*p* < 0.01). In addition, the correlations between the four research variables and the control variables are shown. Age is positively correlated with psychological well-being, employee engagement, personal initiative, and prosocial behavior. Industry type is also positively correlated with psychological well-being, employee engagement, personal initiative, and prosocial behavior. However, job types show significant negative correlation with psychological well-being, employee engagement, personal initiative, and prosocial behavior. Furthermore, there is no correlation between perceived autonomy support and the control variables in this study.

**Table 2:** Descriptive statistics, correlation, and HTMT matrix among variables.

Variable	*M*	SD	1	2	3	4	5	6	7	8
1. Age	39.74	10.07	-	-	-	-	-	-	-	-
2. Job type	6.01	2.98	−0.04	-	-	-	-	-	-	-
3. Industry type	5.80	3.66	0.05	0.07^*^	-	-	-	-	-	-
4. PAS	4.23	1.16	0.04	−0.04	0.03	-	*0.29*	*0.46*	*0.33*	*0.48*
5. PW	4.43	0.58	0.13^**^	−0.09^**^	0.09^**^	0.24^**^	-	*0.63*	*0.67*	*0.62*
6. EE	3.49	0.62	0.32^**^	−0.11^**^	0.08^*^	0.41^**^	0.48^**^	-	0.76	*0.75*
7. PI	3.44	0.60	0.23^**^	−0.12^**^	0.07^*^	0.29^**^	0.52^**^	0.63^**^	-	*0.66*
8. PB	3.24	0.64	0.18^**^	−0.07^*^	0.09^**^	0.43^**^	0.48^**^	0.63^**^	0.56^**^	-

*N* = 1,092. PW, psychological well-being; PAS, perceived autonomy support; EE, employee engagement; PI, personal initiative; PB, prosocial behavior; Values in italics denote a HTMT ratio. ^*^p < 0.05, ^**^p < 0.01.

### 7.2. Validity and reliability analysis

We conducted *CFA* to analyze construct validity and reliability. Construct validity was verified by dividing it into convergent and discriminant validities. Convergent validity was evaluated in terms of the magnitude and significance of the standardized factor loadings (*SFL*) and composite reliability (*CR*). As shown in Table 3, except for autonomy in psychological well-being, the measurement model’s SFL values ranged from 0.51 to 0.88, exceeding the 0.5 standard-cutoff (Hair et al., 2018). Although the autonomy of *SFL* was slightly lower, the value was significant. Furthermore, the *CR* values for all constructs ranged from 0.74 to 0.93, which exceeds the convergent validity threshold of 0.6 (Hair et al., 2018). Therefore, the convergent validity of the measure was appropriate.

**Table 3:** Factor loading and reliability.

Latent variable	Observed variable	*B*	*β*	SE	*t*	*α*	CR
Perceived autonomy support	PAS1	0.86	0.76	0.03	26.24	0.93	0.93
	PAS2	1.09	0.88	0.03	32.35		
	PAS3	0.96	0.82	0.03	31.48		
	PAS4	1.06	0.88	0.03	32.08		
	PAS5	1.06	0.84	0.03	40.72		
	PAS6	1.00	0.81	-	-		
Psychological well-being	Autonomy	0.42	0.34	0.04	9.37	0.79	0.74
	Environmental mastery	0.73	0.63	0.05	14.36		
	Personal growth	1.19	0.76	0.08	14.72		
	Purpose in life	0.83	0.50	0.07	12.07		
	Positive relations with others	0.96	0.58	0.07	13.75		
	Self-acceptance	1.00	0.59	-	-		
Employee engagement	Affective engagement	1.54	0.76	0.08	19.26	0.92	0.78
	Behavioral engagement	1.51	0.78	0.07	20.63		
	Cognitive engagement	1.00	0.65	-	-		
Personal initiative	PI1	0.86	0.66	0.05	19.11	0.86	0.85
	PI2	0.82	0.62	0.05	17.84		
	PI3	0.99	0.71	0.05	20.10		
	PI4	1.05	0.69	0.05	19.74		
	PI5	1.01	0.74	0.05	20.97		
	PI6	0.83	0.55	0.05	17.71		
	PI7	1.00	0.68	-	-		
Prosocial behavior	PB1	1.00	0.65	-	-	0.83	0.83
	PB1	0.82	0.63	0.05	17.36		
	PB2	0.74	0.54	0.05	15.42		
	PB3	0.98	0.73	0.05	20.11		
	PB4	1.07	0.72	0.06	19.52		
	PB5	0.95	0.65	0.05	17.93		
	PB6	0.78	0.56	0.05	16.17		

*N* = 1,092. *B*, unstandardized factor loadings; *β*, standardized factor loadings; SE, standard error; *t*, *t*-value; *α*, Cronbach alpha; CR, composite reliability.

Discriminant validity was tested by comparing the goodness-of-fit between different factor models (Rios and Wells 2014). To determine whether each variable considered in this study was distinct, we performed a number of CFAs. Compared with other models, the proposed five-factor model structure (i.e., psychological well-being, perceived autonomy support, employee engagement, personal initiative, and prosocial behavior) was found to be a significantly better fit for the data (*x*^2^ = 1557.197, df = 350, TLI = 0.91, CFI = 0.92, RMSEA = 0.06, SRMR = 0.06), suggesting that all the variables were distinct from one another. As additional evidence of discriminant validity, we calculated the heterotrait–monotrait (*HTMT*) ratios of the correlations (Henseler et al., 2015), which is an alternative approach to the Fornell–Larcker criterion and the examination of cross-loadings, and is based on the multitrait-multimethod matrix. As a rule of thumb, when *HTMT* is >0.85, discriminant validity poses a problem (Henseler et al., 2015). As shown in Table 1, *HTMT* was calculated at 0.29–0.76, which shows that the constructs had adequate discriminant validity. In conclusion, the discriminant validity of the measures was appropriate.

To assess reliability, we measured Cronbach’s alpha (α) and *CR*. The α values for all constructs ranged from 0.79 to 0.93, which were consistent with Nunnally’s criteria of ≥0.7 (Nunnally and Bernstein, 1994). Furthermore, *CR* values ranged from 0.74 to 0.93, which agreed with Fornell–Larcker’s criteria of 0.6 (Fornell and Larcker, 1981). All other indicators supported the reliability of the construct.

### 7.3. Test of hypotheses

**Figure 3:**
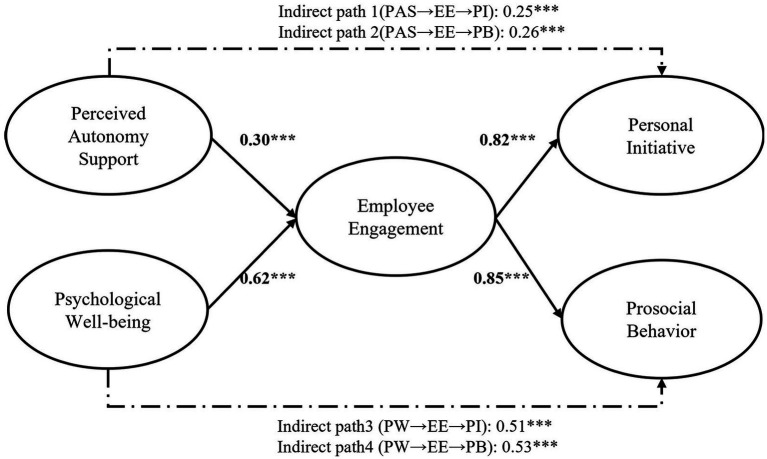
Structural model with standardized coefficients.

We used the structural equation modeling analysis to test the hypotheses. The model examined the effects of job and personal resources on positive performance using perceived autonomy support and psychological well-being as independent variables, employee engagement as a mediating variable, and both personal initiative and prosocial behavior as dependent variables (Figure 3). The results of the research model fit index were good (*x*^2^ = 1911.004, df = 436, TLI = 0.90, CFI = 0.91, RMSEA = 0.06, SRMR = 0.07). Therefore, we assessed direct and indirect effects.

Table 4 shows the results of the analysis of the direct effect relationships between the variables. In line with our hypothesis that perceived autonomy support may be positively associated with employee engagement, the direct path between perceived autonomy support and employee engagement was positive and significant (*β* = 0.30, *p* < 0.001). Thus, H1 was supported. H2 was predicted based on the relationship between psychological well-being and employee engagement. We found support for H2 (*β* = 0.62, *p* < 0.001). H3 postulated the relationship between employee engagement and personal initiative. Employee engagement had a significant influence on personal initiative (*β* = 0.82, *p* < 0.001). H4 assumed that there was a significant relationship between employee engagement and prosocial behavior. This study found support for H4 (*β* = 0.85, *p* < 0.001).

**Table 4:** Results of the path coefficient of the structural model (direct effects).

	*B*	*β*	SE	*t*	*p*
H1: PAS → EE	0.11	0.30	0.01	9.756	0.001
H2: PW → EE	0.41	0.62	0.03	12.173	0.001
H3: EE → PI	1.23	0.82	0.08	16.359	0.001
H4: EE → PB	1.40	0.85	0.09	16.051	0.001

*N* = 1,092. *B*, unstandardized coefficient; β, standardized coefficient; SE, standard error; *t*, *t*-value; *p*, value of *p*.

Given that this model comprised a serial indirect path, an indirect effect test with phantom variables was performed (Chan, 2007). Bootstrapping with a bias-corrected confidence estimate was used to assess the significance of the indirect effects (see Table 5). The results showed that the indirect effect of perceived autonomy support on personal initiative through employee engagement was significant [*B* = 0.15, *β* = 0.25, *p* < 0.001, 95% *CI* (0.11, 0.19)]. Hence, H5 was supported. Similarly, perceived autonomy support had a significant indirect effect on prosocial behavior via employee engagement [*B* = 0.13, *β* = 0.26, *p* < 0.001, 95% *CI* (0.10, 0.16)], thereby supporting H6. Further, the indirect relationship between psychological well-being and personal initiative, mediated through employee engagement, was significant [*B* = 0.50, *β* = 0.51, *p* < 0.001, 95% *CI* (0.41, 0.61)], implying that H7 was supported. Finally, we hypothesized that psychological well-being affects prosocial behavior through employee engagement. The results showed that the indirect effect was significant [*B* = 0.57, *β* = 0.53, *p* < 0.001, 95% *CI* (0.47, 0.69)], thereby supporting H8.

**Table 5:** Results of bootstrapping for the indirect effect of the structural model.

	*B*	*β*	SE	*p*	Boot 95% CI^a^	
					Lower	Upper
H5: PAS → EE → PI	0.15	0.25	0.02	0.001	0.11	0.19
H6: PAS → EE → PB	0.13	0.26	0.02	0.001	0.10	0.16
H7: PW → EE → PI	0.50	0.51	0.05	0.001	0.41	0.61
H8: PW → EE → PB	0.57	0.53	0.05	0.001	0.47	0.69

*N* = 1,092. *B*, unstandardized coefficient; *β*, standardized coefficient; SE, standard error; *p*, value of *p*. ^a^Bootstrapping re-sampling 5,000 and bias-corrected 95% CI.

## 8. Discussion

Although the degree of impact depends on the occupational group, the pandemic significantly affected workplace health and safety, which resulted in drastic changes in the work environment, including telecommuting (Rudolph et al., 2021). This led to a number of studies on psychological capital focusing on employee health and well-being (e.g., burnout, life satisfaction, loneliness, and procrastination; Van Roekel et al., 2021; Wang et al., 2021) and performance (e.g., job performance; Bakker et al., 2021; Wang et al., 2021). In this context, we aimed to investigate the relationship between variables influenced by rapid changes in the work environment represented by the COVID-19 pandemic as a major social crisis. Furthermore, we examined how job autonomy influences in-role performance in the form of proactive employee characteristics, as well as extra-role performance in the form of prosocial behavior, as mediated by employee engagement, using the job demands-resources model.

Our findings can be summarized as follows. First, job autonomy as a job resource has a statistically significant positive effect on employee engagement. This result supports previous findings that job resource components such as feedback, autonomy, and task significance have a positive effect on job engagement (Schaufeli and Bakker, 2004; Demerouti and Cropanzano, 2010; Cho and You, 2020; Choi et al., 2020). Of the factors eliciting job engagement and high job performance, job autonomy has drawn attention as a critical antecedent (Bakker and Geurts, 2004; Hakanen and Roodt, 2010; Anderson et al., 2014); in the job demands-resources model (Demerouti et al., 2001; Bakker and Demerouti, 2008), job resources, such as job autonomy, are assumed to influence job engagement through motivation. Recent meta-analyzes have shown that job autonomy positively affects job engagement among employees (Crawford et al., 2010; Christian et al., 2011; Lesener et al., 2019). These results are consistent with our findings.

Second, employees’ psychological well-being as psychological capital was found to have a statistically significant positive effect on employee engagement. This finding is in line with research showing that employees with higher psychological capital tend to change their thinking and attitudes toward their organization in a positive direction, have a higher sense of belonging, are self-motivated to learn, and adjust to changes more appropriately (Lim and Kim, 2019; Shin and Kim, 2020). This is also consistent with the finding that psychological capital promotes active job participation and a tendency to innovate to improve job performance (Luthans et al., 2007; Jeong et al., 2011). This also supports the finding that psychological capital positively influences employees’ job happiness, job satisfaction, and engagement (Youssef-Morgan and Luthans, 2007; Salanova et al., 2011).

Third, employee engagement positively influences job performance in a statistically significant way. This finding supports the results of previous studies that job engagement, presented by Kahn (1990) as a positive psychological factor that induces work motivation, is an important factor in generating positive outcomes in areas such as productivity (Masson et al., 2008; Rich et al., 2010) and overall job performance (Bakker and Demerouti, 2008; Rich et al., 2010; Kartal, 2018). The significance of this finding can be confirmed and explained by previous studies on the positive effects of employee engagement on job performance (Baterman and Strasser, 1984; Meyer and Allen, 1991; Nasurdin et al., 2018).

Fourth, employee engagement was found to have a significant mediating role with performance variables, when focused on aspects of employee engagement as the degree to which employees feel enthusiasm for their work, promote their dedication and their own work with autonomous efforts in the organization. In other words, it was confirmed that employee engagement is significant as a mediating factor that manifests innovative behavior, and it is consistent with the results of previous studies that employee engagement can promote and mediate innovative behavior of members in an environment with high job autonomy and satisfaction with basic psychological needs (Karatepe et al., 2013; Kim, 2017; Kartal, 2018; Kwon and Kim, 2020; Kim and Song, 2022).

Job autonomy and emotional stability, through psychological well-being, contribute to improvement in work performance by promoting employee engagement, thus, allowing employees to proactively perform jobs and actively cooperate with others. Therefore, companies that are experiencing rapid work environment changes through digital transformation, such as telecommuting and online work cooperation after the COVID-19 pandemic, need to pay more attention to the importance of highly motivating jobs and the influence of psychological resource factors (Rudolph et al., 2021; Demerouti and Bakker, 2023).

### 8.1. Theoretical and practical implications

Based on these results, the following theoretical implications are proposed. Using the job demands-resources model, we identified job autonomy and psychological well-being as the variables most influenced by changes in the work environment during the COVID-19 pandemic. We found that job autonomy and psychological well-being positively influence job performance by improving employee engagement (Brown, 1996; Binnewies et al., 2008; Macey and Schneider, 2008; Leiter and Bakker, 2010; Shuck, 2011; Schaufeli and Tarris, 2014; Laschinger et al., 2015; Zhao et al., 2015). Recently, the job demands–resources model has been expanded to include personal resources. Personal resources refer to an individual’s sense of having the ability to successfully control and influence their environment (Xanthopoulou et al., 2009). Personal resources include self-efficacy, optimism, and self-respect in relation to the organization; they are variable, activated by job resources, situation sensitive, and influence job engagement (Bakker and Demerouti, 2008; Demerouti and Bakker, 2023). The study results contribute to the empirical verification of this theory by confirming the influence of well-being as a psychological perception of personal resources.

In addition, this study has practical implications for human resource management and development practitioners. First, it is important to find concrete measures to enhance the psychological well-being of employees as a psychological micro-foundation to raise productivity and performance in the workplace in the global market, where human resources are a source of competitive advantage and technological environment is rapidly changing. Psychological well-being, as a psychological variable, is strongly related to employees’ attitudes and performance. Previous studies have found that psychological capital can be improved to a certain degree with short-term learning or training, but it is necessary to prepare specific measures. Instead of creating and providing a uniform training program, it is necessary to establish a learning system in the workplace that provides a variety of job experiences. Second, to maintain and improve the level of employee engagement at the organizational level, organizations should give more meaning to the work of employees and support them in maintaining a positive emotional state. It is also important to create an atmosphere in which employees are aware of the company’s situation, become more engaged, and behave in an innovative way. It was proven that the higher the level of employees’ self-initiated awareness of their work, the higher the level of employee engagement and job performance. If employees recognize that their work permits self-realization, with the support of job resources they will be able to have fun and feel alive while working. Ultimately, this will contribute to personal growth and organizational performance. In addition, the importance of prosocial behavior influenced from employee engagement, which believed that facilitates the effective functioning of the organization, has been raised from this study. As a method of motivating direct social relations behavior, a reward system for prosocial behavior can be suggested, such as providing paid vacation or activity support expenses for regular community activities.

### 8.2. Limitations and recommendations for future research

This study serves as a useful baseline for further investigation; however, it has several limitations. First, it depends on a self-reported questionnaire and uses convenience sampling among employees of several Korean companies, which may have led to a sampling bias. Therefore, it is necessary to conduct further research using a large sample with multinational demographics, diverse industrial characteristics, and cultural backgrounds to increase generalizability. In addition, to overcome the limitations of self-report surveys, various measurement methods must be employed to increase objectivity in the case of job performance variables.

Second, job autonomy, as a job resource, and psychological well-being, as a personal resource, were selected as independent variables to reflect the impact of COVID-19. In subsequent studies, other resources should be considered, including familial and environmental resources; emotional support from one’s family greatly influences one’s personal psychological well-being (Kalliath et al., 2019). Strengthening of organizational communication policies should be considered, as their influence on promoting the psychological stability of employees has become more important because of the COVID-19 pandemic (Tuan, 2021; Demerouti and Bakker, 2023).

Lastly, many studies in the field of JD-R theory or conservation of resources theory have considered rotation intention as an important output variable along with work performance and have tried to confirm the influence of individual-level variables (Schaufeli and Bakker, 2004; Halbesleben, 2010; Shuck, 2011). In this study, however, the range of resources that have been discussed so far has been expanded to include interpersonal relationships within organizations and suggestions have been made to consider the influence of these variables on work performance through employee engagement. In future studies, based on the extensibility of these theories, it is suggested to examine the empirical relationship between the psychological well-being and employee engagement, which are the psychosocial variables presented in this study, and the improvement of the psychological aspect of job performance such as a lower turnover rate.

### 8.3. Conclusion

This study aimed to investigate the relationship between psychological well-being, job autonomy, and in-role (personal initiative) and extra-role performance (prosocial behavior) mediated by employee engagement. It found that job autonomy and psychological well-being (as personal resources), influence job performance–personal initiative and pro-social behavior—through improvements in employee engagement. Our results highlight the importance of employee engagement as a psychological micro-foundation for employees and the roles of personal initiative and prosocial behavior in times of rapid changes in the work environment due to the pandemic.

## Data availability statement

The raw data supporting the conclusions of this article will be made available by the authors, without undue reservation.

## Ethics statement

Ethical review and approval was not required for the study on human participants in accordance with the local legislation and institutional requirements. The patients/participants provided their written informed consent to participate in this study.

## Author contributions

DL: wrote and revised the manuscript, funding acquisition. YJ: designed the study, analyzed the data and revised the manuscript. All authors contributed to the article and approved the submitted version for publication.

## Funding

This study was supported by the University of Suwon (2021).

## Conflict of interest

The authors declare that the research was conducted in the absence of any commercial or financial relationships that could be construed as a potential conflict of interest.

## Publisher’s note

All claims expressed in this article are solely those of the authors and do not necessarily represent those of their affiliated organizations, or those of the publisher, the editors and the reviewers. Any product that may be evaluated in this article, or claim that may be made by its manufacturer, is not guaranteed or endorsed by the publisher.
